# From Creation to Consolidation: A Novel Framework for Memory Processing

**DOI:** 10.1371/journal.pbio.1000019

**Published:** 2009-01-27

**Authors:** Edwin M Robertson

## Abstract

Long after playing squash, your brain continues to process the events that occurred during the game, thereby improving your game, and more generally, enhancing adaptive behavior. Understanding these mysterious processes may require novel theories.

Long after playing a game of squash or reading this essay, your memory for playing and reading continues to be processed by your brain. These “offline” processes improve your game and your understanding of this essay, and more generally, enhance adaptive behavior. Yet progress in understanding how the brain regulates the offline processing of memories has been hampered by the absence of robust models for interpreting diverse, and often contradictory, experimental results.

In the last 20 years, highly fertile quantitative models across the biological spectrum from the molecular to the behavioral have proved critical in advancing our understanding of memory encoding (e.g., [1–5]). But these models have focused upon the exact moment a memory is formed; while our ability to recall an event is dictated, at least in part, by events that precede and follow the encoding of a new memory. The critical role that events following memory encoding play in determining subsequent recall have been recognized for at least the past 100 years [6]. Yet few, if any, models have been formulated for these “offline” processes that produce qualitative and quantitative changes in a memory during consolidation (Box 1). Our attempts to understand these mysterious processes have generated a purely descriptive set of observations. Although these observations have provided critical glimpses into offline memory processing, they have also produced unresolved contradictions between some of the most fundamental and critical sets of observations (for reviews, see [7–11]).

**Box 1. Memory Consolidation**

A memory passes through at least three key milestones in its development: initially it is encoded, then it is consolidated, and finally it is retrieved. During consolidation a memory can undergo both quantitative and qualitative changes. A memory may be enhanced, demonstrated by a quantitative increase in performance, or it may be stabilized, demonstrated by becoming quantitatively less susceptible to interference [10,46,47]. A memory can also undergo qualitative changes: there can be a shift in the strategy used to solve a problem or the emergence of awareness for what had earlier been learned [49,50]. Although there is a rich diversity in the behavioral expression of consolidation, each of these examples may rely upon the same underlying computation (see main text). Consolidation is measured as a change in performance between testing and retesting [46,47]. Contrasting final performance at retesting against an initial baseline provides a direct measure of “offline” performance changes that occur during consolidation.

For example, one set of observations suggests that consolidation may occur over any time interval, whereas another body of data suggests that these processes require sleep [6,8]. Clearly, both cannot be true. Resolving the inherent conflict between these perspectives strikes at the very heart of how biological mechanisms process memories after their initial encoding. Making sense of what threatens to become an avalanche of disconnected and incoherent empirical findings may require novel theories that can simultaneously reconcile apparently inconsistent observations and provide a fertile, hypothesis-driven framework for future work. Here, drawing upon examples mainly from the processing of motor skill memories, I take the first tentative steps toward assembling such a framework.

## Toward a Unifying Framework

### Distinct circuits operate during sleep and wakefulness.

**Figure 1:**
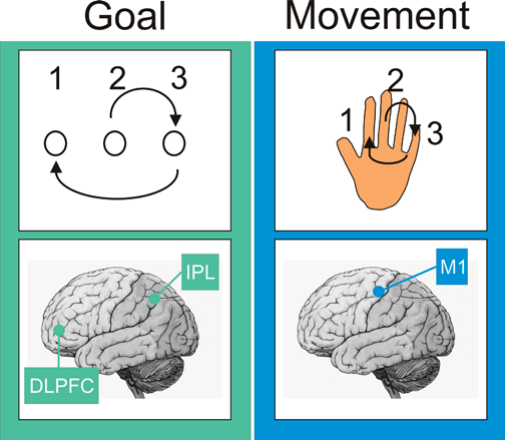
A Motor Skill Memory Has Classically Been Split into Two Components One component encodes the spatial goal of the movement, and the other encodes the movements needed to achieve that goal [84–86]. For example, the goal of playing out a sequence of spatial positions—2-3-1—can be achieved by a sequence of finger movements. The goal of a motor skill is encoded within a circuit that includes the dorsolateral prefrontal cortex (DLPFC), the inferior parietal lobule (IPL), and perhaps the mediotemporal lobe (MTL); whereas the movements associated with a skill are encoded within a circuit that includes the primary motor cortex (M1) and subcortical areas such as the striatum [12,13]. Other memories can be split into similar components. For example, navigating around a city relies upon learning the spatial location of landmarks plus learning the sequence of right-and-left turns needed to get to the landmark.

**Figure 2:**
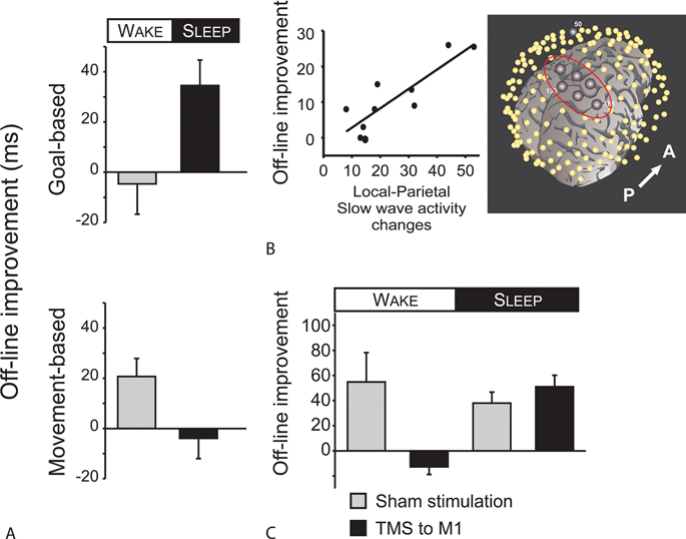
Motor Skill Memories Are Differentially Processed over Wakefulness and Sleep (A) The spatial goal of a motor skill is processed over sleep but not during wakefulness, whereas the skilled movements are processed over wakefulness but not over sleep [15]. This double dissociation implies that distinct mechanisms are engaged to support consolidation over wakefulness and sleep. The offline processing of memories during consolidation can be expressed as an offline increase in motor skill [8,46]. (B) Using high-density EEG, the parietal cortex has been implicated in supporting motor skill improvements over sleep [20]. The high-density electrodes (yellow dots) were aligned with a magnetic resonance image of a participant's brain. Following motor skill learning, a cluster of electrodes (white dots, enclosed by a red circle) centered over the parietal cortex showed an increase in slow-wave activity during sleep. (C) In contrast, a circuit that includes M1 makes a critical contribution to the consolidation of motor skills over wakefulness but not over sleep [16–18]. Disruption of M1, by applying TMS, blocks the development of motor skills over wakefulness but not over sleep.

**Figure 3:**
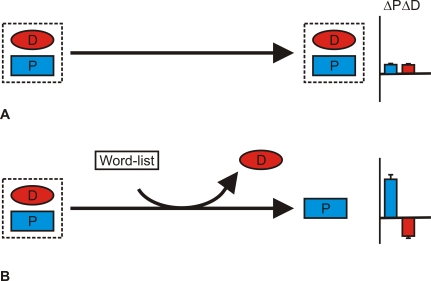
Memory System Interactions during Consolidation (A) Our behaviors are frequently supported by a blend of declarative (D) and procedural (P) knowledge. In such behaviors, procedural (ΔP, blue bar) and declarative (ΔD, red bar) knowledge change little over wakefulness. (B) When the declarative component of such behaviors is knocked out—for example, by learning an interfering word list—there is a substantial enhancement in motor performance. This implies that declarative knowledge inhibits motor consolidation over wakefulness [48].

Important clues about the offline processing of memories can be gleaned from understanding how the brain initially encodes memories. The motor skill memories acquired by a squash player, for example, ensure the production of rapid and powerful arm movements, either backhand or forehand, to hit a ball. The goal is always the same—to hit a ball; however, the exact movements can be very different. This classical distinction between goal and movement can be mapped onto distinct brain circuits (Figure 1, [3,12,13]). These distinct circuits are differentially affected by wakefulness and sleep: activity within the goal-component circuit changes substantially between wakefulness and sleep, with far smaller changes in activity occurring within the movement-based circuit [14]. Having differential changes in activity may produce a differential processing of the motor skill components during wakefulness and sleep. Consistent with this idea, experimental work has shown that only the movement component is processed during wakefulness; whereas only the goal component is processed during sleep (Figure 2, [15]). Converging with this behavioral work are observations from functional imaging and transcranial magnetic stimulation (TMS) studies showing that distinct circuits are engaged during wakefulness and sleep to support offline processing (Figure 2, [16–20]).

Recent functional imaging work has shown that the primary motor cortex (M1), which is associated with movement-based processing, and parietal areas, which are associated with goal-based processing, are both activated after motor skill learning [19]. But subsequent consolidation is limited to being either goal- or movement-based [15,21]; suggesting that the offline activation of brain areas, alone, is not sufficient to support consolidation. Movement-based consolidation is dependent upon M1, and communication within that small local circuit is facilitated by high-frequency oscillations that are prominent during wakefulness [16–18]. In contrast, goal-based consolidation may be dependent upon communication across a large circuit including the parietal and prefrontal cortices, which is facilitated by slow-frequency oscillations that are a hallmark of nonrapid eye movement (NREM) sleep (Box 2, [22–24]). Thus multiple circuits may remain activated after learning; but because of the properties of specific brain states (e.g., wakefulness versus NREM sleep), only one of these circuits may make a functional contribution to subsequent consolidation.

**Box 2. Mammalian Sleep**

There are two broad types of sleep: NREM sleep and REM sleep. At the onset of sleep, humans enter the first of the four stages (1 to 4) of NREM sleep. As sleep deepens, stage 1 progresses into stage 4 and there is a decrease of electroencephalographic (EEG) frequency [62]. Each of the NREM stages is defined by arbitrary criteria. For example, stage 2 sleep is defined by K-complexes (large, sharp waves on the EEG) and sleep spindles (12–14 Hz bursts of synchronized EEG activity). Increasingly, experimental work has attempted to link these electrophysiological features, as opposed to the sleep stages they define, to specific functions; for example, fast and slow sleep spindles may be linked to different aspects of memory processing [63]. The final two stages of NREM sleep, stages 3 and 4, are characterized by the increasing predominance of low-frequency oscillations (<4 Hz). These oscillations travel slowly across the cortex, earning these latter two sleep stages the combined term of slow-wave sleep (SWS). REM sleep follows a bout of NREM sleep, and is characterized by desynchronized, high-frequency activity similar to the pattern of activity during wakefulness [64]. During REM sleep there is a functional paralysis of many of the skeletal muscles; however, bursts of rapid eye movements are still possible and earn this sleep stage its name. Along with these electrophysiological changes, there are also dramatic changes in neurochemistry as the brain passes from NREM into REM sleep [65]. Blocks of NREM-REM appear throughout the night and last for approximately 90–100 minutes before another NREM-REM cycle begins. The length of the cycle remains roughly constant throughout the night; however, its composition changes with SWS dominating early in the night; while REM comes to dominate later in the night.

Many recent functional imaging studies have sought to provide insight into the neural basis of consolidation. These studies have contrasted the patterns of activation before and after consolidation to reveal how the brain has been changed by consolidation [25–28]. Yet just because the activation of a brain area is changed by consolidation does not mean that area was responsible for supporting consolidation [29]. For example, changes within one brain area may have been driven by another brain area. To reveal the brain areas supporting consolidation, it is first necessary to identify those brain areas activated during consolidation (e.g., [19,20]). Disrupting the function of these activated areas, by using TMS or through lesion studies, would determine which areas are necessary for consolidation [30]. Thus, the challenge for future studies is to identify those circuits activated during consolidation, as opposed to those circuits altered by consolidation, and use this as a foundation to define those circuits making a functional contribution to consolidation.

Our declarative memories—those corresponding to facts and events—may also be processed offline through distinct mechanisms (Box 3). For example, when only a single word list is learned, the subsequent offline processing occurs during both sleep and wakefulness [31]. In contrast, when an association is learned between list of words, the subsequent processing takes place only during sleep [32,33]. So one mechanism, engaged during wakefulness, supports consolidation when there are no associative links among a list of words, and another distinct mechanism, engaged during sleep, may support consolidation when there are associative links between declarative memories. Recent work has shown that the retrieval of a simple list of words is enhanced after sleep [34]. However, retrieval is influenced by many factors, only one of which is memory consolidation (Box 1). Future studies might determine whether the enhanced retrieval of a simple word list after sleep is due to sleep-dependent consolidation.

**Box 3. Memory Classification**

Human memories have been classified into two broad types: declarative memories, dealing with memories for facts and events, and procedural memories, dealing with memories for skills [1]. Overlying this classification is another classification distinguishing between memories that individuals are aware of acquiring (explicit memory) and unaware of acquiring (implicit memory). These different classifications should be seen as being largely independent. We can be aware of acquiring a new skill; for example, learning to ride a bike (explicit-procedural), but it is also possible to be unaware of acquiring a new skill, as occurs for the grammatical rules we learn (implicit-procedural). Similarly, we are aware of learning a new set of terms (explicit-declarative) but unaware that subliminal advertising, or priming in a psychology experiment, may affect our selection of a brand, or cause us to declare that we have already seen a list of words (implicit-declarative). The declarative-procedural classification has been mapped onto specific neural circuits: the mediotemporal lobe (MTL) supports declarative memories while motor cortical areas and subcortical areas, including the striatum and cerebellum, support procedural memories [1]. This concept has been challenged by recent work showing that the MTL, at times, makes important contributions to procedural learning [25,37,56,66]. Rather than constituting part of a declarative memory system, the MTL may support a set of computations that are important to both declarative and procedural memory processing [67].

A differential organization—with distinct mechanisms engaged over wakefulness and sleep—is highly flexible, and may allow memories to be processed offline across a wide variety of different situations. When both sets of mechanisms are engaged, offline memory processing can occur over any time interval; but when one or the other mechanism is inhibited, processing will be restricted to either sleep or wakefulness. A differential organization, therefore, provides a unique framework for explaining the rich variety of contexts in which offline memory processing has been observed to occur. Controlling the flow of information through this framework may be achieved, at least in part, by the effects of practice and memory system interactions.

### Practice and its effects on consolidation.

A high degree of flexibility can arise from a differential organization when the distinct sets of mechanisms are independently controlled. One way for independent control to be achieved is for practice to engage the distinct mechanisms in an asymmetric manner. For example, short bouts of practice can produce a motor skill memory with a large goal-based and minimal movement-based component, while prolonged practice periods may produce the reverse [3,13]. These motor skill components are processed offline over different brain states (i.e., sleep versus wakefulness), and so differences in the relative size of these component may restrict the benefits of consolidation to a specific brain state [15]. Should the larger component be movement-based, then the benefits of consolidation may develop only during wakefulness; if the larger component is primarily goal-based, then consolidation may occur only during sleep. Such a prediction assumes that any component is sufficiently large to trigger consolidation [35]; but not so large that any possible benefits of consolidation have already been achieved through practice ([36]; e.g., great skill may have already been acquired with practice, leaving little opportunity to enhance skill further during consolidation). Thus, by altering the relative proportions of motor skill components, practice may determine whether the benefits of offline processing are predominately wake- or sleep-dependent.

Rate of skill acquisition may also alter the relative proportions of motor skill components, with fast learning favoring the acquisition of goal-based improvements and leading to improvements that are predominately sleep-dependent ([37]; see also [35,38]). Similarly, task properties may alter the relative proportions of the motor skill components [39,40]. For example, we communicate face-to-face by learning to articulate a language while also learning a set of nonverbal cues such as hand gestures, which provide important contextual cues to our spoken words [41]. Having a contextual element embedded within a task shifts the circuits responsible for supporting motor skill learning to favor those implicated in goal-based learning [39,41]. Potentially, this leads to motor skill acquisition that is predominately goal-based, and as this component is preferentially processed over sleep, to a task that shows sleep-dependent consolidation ([39]; see also [40,42]). Thus the extent of practice and task properties, by altering the relative proportions of motor skill components, may determine whether consolidation occurs over any time interval or is specifically dependent upon wakefulness or sleep.

### Interactions across memory systems may support offline processing.

Many behaviors are supported by a combination of motor skill and declarative knowledge; for example, skillfully tapping out and knowing your personal ID number to get cash from a machine [43]. In contrast, many other behaviors—such as exercising social judgment, applying grammatical rules, or using intuition—require little or no declarative knowledge [44]. When a motor skill and declarative knowledge are acquired simultaneously, the subsequently offline motor processing depends on sleep; whereas, when a motor skill is acquired with little or no declarative knowledge, the subsequent offline motor processing occurs during wakefulness or sleep [39,45]. So when skills are acquired along with declarative knowledge there is little offline motor skill processing during wakefulness, which implies that declarative knowledge may block offline motor processing.

Two important predictions flow from this hypothesis. When a motor skill is acquired without declarative knowledge—as occurs during implicit learning—the subsequent offline processing of the motor skill should be blocked by declarative learning (Box 3, [31]). This idea was tested by having participants acquire a motor skill, declaratively learn a list of words, and later have their skill retested. Motor skill decreased between testing and retesting in proportion to the amount of prior declarative learning [31]. The decrease in motor skill implies that the offline mechanisms, which normally support maintenance or enhancement of motor skill, are blocked by declarative learning [46,47]. These observations are consistent with the hypothesis that declarative knowledge blocks offline motor processing.

When a motor skill is acquired along with declarative knowledge—as occurs during explicit learning—removing or “knocking out” the declarative memory should induce offline motor memory processing, leading to enhanced performance (Box 3 and Figure 3). Consistent with this prediction, when declarative knowledge for a previously acquired motor skill was knocked out, participants' motor skill was enhanced [48]. Disrupting declarative knowledge for the motor sequence was achieved by having participants learn a list of words. The declarative knowledge for the list of words interfered with and so reduced participants' declarative knowledge for the 12-item sequence from 7.3 ± 0.9 to 4.0 ± 0.8 items. In principle, any intervention that disrupts declarative knowledge should result in the offline enhancement of motor skill. In contrast, when declarative knowledge for the motor skill is not disrupted, there is no enhancement of motor skill. Thus, interactions occurring between memory systems can play an important role in controlling the processing of memories after their acquisition.

### Disengagement of memory systems during sleep.

During wakefulness, reciprocal interactions occur between memory systems; whereas during sleep these systems operate independently [31,45,48]. For example, declarative learning can block the consolidation of motor skills during wakefulness but not during sleep [31]. Likewise, motor skill learning can block the consolidation of declarative memories during wakefulness but not during sleep [31]. Such observations may specifically arise from a reciprocal interaction between the movement component of a motor skill memory, which is processed during wakefulness, and a declarative memory [15,21]. Overall, these observations show that reciprocal interactions occur between memory systems during wakefulness, but that these systems operate independently during sleep.

**Figure 4:**
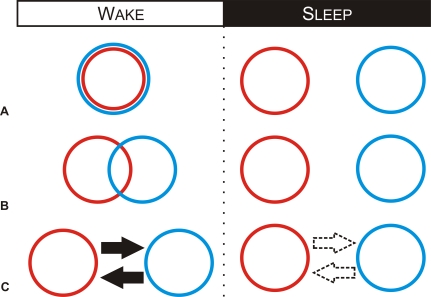
A Schematic Showing How Memory Systems May Interact during Wakefulness, and Operate Independently during Sleep

The transformation from interactive to independent processing implies that the memory systems disengage during sleep, through various potential mechanisms (Figure 4 in Box 4), allowing the simultaneous processing of both procedural and declarative memories [31,49]. Aspects of disengagement can be replicated during wakefulness by removing the inhibitory influence of declarative knowledge and allowing the consolidation of motor skills (Figure 3, [48]). Thus, offline memory processing may involve not only engaging specific neuroplastic mechanisms, but also disengaging interacting memory systems.

**Box 4. Mechanisms of Disengagement**

Several mechanisms can explain how memory systems interact during wakefulness and operate independently during sleep. (A) The same neuronal resources may support procedural (blue) and declarative (red) processing during wakefulness; whereas distinct resources may support memory processing during sleep. (B) Alternatively, declarative and procedural processing may partially share resources during wakefulness but not during sleep. For example, some brain areas, such as the MTL, support the processing of both declarative and procedural memories [37,56,66]. Having shared neuronal resources during wakefulness may account for the reciprocal interactions between the declarative and procedural memory systems. (C) Finally, sleep may functionally disconnect the declarative and procedural systems, allowing them to operate as independent memory systems. Combinations of these broad mechanisms are also possible: for example, overlapping neuronal resources may become functionally disconnected during sleep. These different scenarios make unique sets of predictions; consequently, future work should be able to distinguish among them.

Disengagement may increase the computational power of memory processing during sleep by removing the interfering effects of memory system interactions. It is during sleep that the brain's capacity to reorganize information and reveal “hidden patterns” becomes particularly marked (Box 5). This greater ability to discover hidden patterns may underlie our intuitive sense that “sleeping on a problem” can produce a solution. For example, a mathematical problem can be solved either by systematically working through a series of intermediary steps to calculate the final solution, or by discovering a hidden pattern and seeing that one of the early steps predicts the final solution [50]. An individual's capacity to bypass the intermediary steps increases following sleep [50] and requires the formation of an association between one of the early steps and the final solution. Forming associations between temporally distant events occurs readily during sleep [50,51] and may depend upon placing small fragments of juxtaposed events in the correct temporal order, and then fusing those events together. For example, a sequence of items may be recalled as a series of short fragments, such as 2-1-2, 2-3, and 3-4. After a night's sleep, these fragments may be fused together to form 2-1-2-3-4 [49]. Forming these high-order associations may allow memories that have been disrupted during the day to be reconstructed during sleep [36,52]. Such high-order processing can occur within the declarative memory system—expressed as enhanced declarative recall [49]—and within the procedural system—expressed as improved motor performance [39,45,53–55].

**Box 5. Mechanisms of Engagement**

Although disengagement may be necessary for offline memory processing, it is unlikely to be sufficient: as some mechanisms are disengaged, other mechanisms will become engaged. Two prominent theories describe the mechanisms that may be engaged. One theory focuses on increasing the signal associated with a memory by replaying past experiences [68]. Consistent with this theory, neuronal activity patterns associated with the performance of an earlier behavior are played out again during an offline interval [69,70]. Such neuronal reactivation is a common feature of offline activity and has been found within many brain areas and across many brain states [69–73]. Thus, neuronal reactivation occurs within brain areas, such as the hippocampus, that are associated with memory processing, and over intervals that are known to support offline processing.

An important challenge for future work is to test the link between neuronal reactivation and offline memory processing. Recent studies have started to address this challenge. Offline performance changes are correlated with neuronal reactivation [74,75], and these offline performance changes can be increased by increasing neuronal reactivation [76]. These latter observations suggest a causative link between neuronal reactivation and offline performance improvements [76]. A critical future test for the neuronal replay theory will be to establish that disrupting neuronal reactivation can prevent offline performance changes.

An alternative theory, the synaptic homeostasis theory, suggests that rather than increasing the signal associated with a memory, offline processing may reduce the noise associated with a memory. During wakefulness, much of the increase in the efficacy and number of synapses [77,78] may be driven by chance events that add noise to a network by obscuring synaptic changes driven by predictable events that can guide adaptive behavior. Removing noise-related synaptic changes is thought to be the function of SWS [77,78]. Synaptic changes—driven by learning or the induction of neuroplasticity—have been convincingly linked to SWS [20,79,80]. Yet the nature of this link, and specifically whether it is due to SWS reducing the efficacy of a specific population of synapses, is poorly understood. It is conceivable that both of these mechanisms operate together—one improving the signal of a memory, the other decreasing the noise—to mediate offline memory processing.

Generation of high-order associations has been linked to the hippocampus, a brain area frequently implicated in sleep-dependent processing [11,25,39,56]. The brain's greater affinity to generate high-order associations during sleep may stem, at least in part, from disengaging the memory systems. Yet disengagement is unlikely to explain all aspects of memory processing over sleep (Box 5). Although disengagement may increase the capacity for processing within each memory system by decreasing the potential for interference between the systems, this independence comes at the cost of impairing integration across memory systems. Disengagement, and its associated costs, may be restricted to a specific stage of sleep (e.g., NREM; Box 2); while other brain states, including other stages of sleep (e.g., rapid eye movement [REM] sleep; Box 2) and wakefulness, support a more interactive mode of processing. Competition for access to these brain states, and their associated interactive and disengaged modes of processing, could produce a diverse range of processing individually tailored to each memory. When interactive processing dominates, the benefits of consolidation may be restricted to a single memory system [49,50]; alternatively, the benefits of consolidation may be seen across both memory systems, a feature of disengagement [31].

The disengagement between memory systems during sleep may be due to changes in functional connectivity. During wakefulness, there is a reciprocal dialogue between the hippocampus and cortical areas; in contrast, during NREM sleep (Box 2), communication appears unidirectional, from the cortex to the hippocampus [57,58]. These changes in connectivity may impair the communication between memory processing areas within the hippocampus and cortex, which may lead to memory system disengagement. Changes in cortical connectivity have also been revealed in recent studies using TMS. A TMS pulse can propagate (a measure of connectivity) less during NREM sleep than during wakefulness [59]; however, the distance of propagation depends upon the site of stimulation, with TMS applied to areas lying anterior to M1 producing a short-distance pulse and TMS to more posterior areas (e.g., sensorimotor areas) producing a long-distance pulse [60], similar to that seen during wakefulness. Thus, some brain areas appear to become functionally isolated during NREM, due to diminished connectivity, while other areas may remain as functionally connected as they were during wakefulness.

These heterogeneous changes in functional connectivity during NREM sleep may support reduced connectivity between memory systems, allowing disengagement, while simultaneously supporting enhanced or maintained connectivity within memory systems, allowing the offline processing necessary for memory consolidation. Alternatively, a decrease in functional connectivity may be associated with a specific sleep stage—such as NREM—while other sleep stages support the offline processing within specific memory systems. This alternative implies that when declarative and procedural memories are acquired simultaneously, consolidation will be dependent upon NREM sleep when memory systems are disengaged. In contrast, when memories are acquired in isolation, consolidation will not depend on NREM sleep because it will not require disengagement. Consistent with this prediction are observations that the consolidation of motor skills, when acquired along with declarative knowledge for the skill, is correlated with NREM sleep [45], whereas when the same motor skill is acquired in isolation, its subsequent consolidation is correlated with REM sleep [15]. Thus, a single sleep stage or the combined action of several sleep stages may be responsible for coordinating both the engagement and disengagement between memory systems.

Evidence that the memory processing benefits of sleep can be replicated over wakefulness through the loss of declarative knowledge ([48], Figure 3) implies that the loss of declarative knowledge may be critical for memory processing during sleep. Yet the sleep-related memory processing benefits can occur without a permanent loss of declarative knowledge. Rather than a permanent loss, sleep may provide a functional loss of knowledge by making a memory temporarily inaccessible to other systems [61]. Thus, sleep may provide an environment in which it is possible to forget a memory, permanently or temporarily, to facilitate processing of other memories.

The interactive and independent modes of memory processing observed during wakefulness and sleep are complementary because each one compensates for the disadvantages of the other [31,48]. Interactions between memory systems can allow knowledge within one system to inform and guide the attainment of knowledge within another system. However, reciprocal interactions between memory systems are vulnerable to interference, such that consolidation within one system can block consolidation within the other [31]. Memory systems operate independently during sleep, mitigating the problem of interference between the systems. But independence produces its own problem by not allowing the integration of information across memory systems, and this is mitigated by the interactive processing during wakefulness. Thus, the proposed framework may have been selected through evolution to support diverse and complementary modes of memory processing—providing the benefits of integration and independence.

## Concluding Remarks

**Figure 5:**
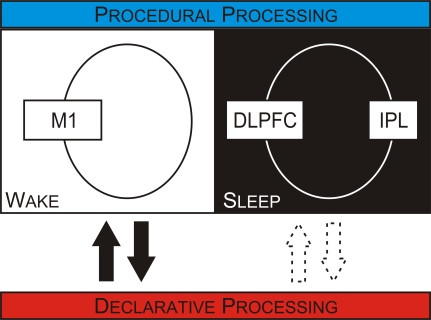
Distinct Mechanisms Are Engaged To Support the Offline Processing of Motor Skill Memories over Wakefulness and Sleep One set of mechanisms, engaged over wakefulness, is supported by a circuit that includes the M1. In contrast, over sleep, a different circuit that may include the prefrontal and parietal cortices is engaged to support motor skill consolidation. Distinct mechanisms that are differentially engaged over sleep and wakefulness may also be responsible for the consolidation of perceptual and declarative memories. Overlying this differential organization within memory systems are reciprocal interactions between memory systems. Declarative processing can block procedural consolidation, and the reciprocal relationship also occurs, with procedural processing blocking declarative consolidation. These interactions are present over wakefulness (solid arrows) but not over sleep (outline arrows). DLPFC, dorsolateral prefrontal cortex; IPL, inferior parietal lobule.

The concepts presented here allow many contemporary observations to be reconciled within a single unified framework (Figure 5). Yet this unique framework extends beyond accounting for observations by also making experimentally testable predictions for future work (Box 6). Direct evidence for a differential organization supporting the offline processing of declarative memories is awaited, and a greater understanding of the relationship between biological events, such as decreases in functional connectivity and the disengagement between memory systems during sleep, is required. Such work will inevitably challenge and perhaps falsify the framework. But the utility of the framework lies within its potential to steer this fledgling and recently rejuvenated field away from the quagmire of disconnected findings and toward more fertile pastures. By so doing, it will help illuminate our understanding of the processing beyond the moment of memory creation, into how memories are consolidated, and so extend our appreciation for how we adapt to this ever changing world.

**Box 6. From Principles to Predictions**

Two important principles lie at the heart of this framework. The first proposes that distinct mechanisms—one operating during wakefulness and the other operating during sleep—are responsible for the continued processing of a memory after its encoding. This implies that different memory components are processed during wakefulness and sleep, and that distinct circuits support memory processing during wakefulness and sleep. These predictions have, in part, been examined for the processing of motor skills [15,18,20,43]. But whether this principle also applies to the processing of perceptual skills and declarative memories has yet to be examined. For example, the primary visual cortex may be responsible for processing perceptual memories during wakefulness, whereas higher visual areas would be responsible for processing these memories during sleep [81–83].

The second principle proposes that memory processing during sleep requires both the engagement of neuroplastic mechanisms as well as the disengagement of interactions between memory systems. This principle can be used to forge a mechanistic link, which is discussed more fully within the main text, rather than just an empirical mapping between memory processing and sleep architecture. Disengagement may not only lead to different aspects of sleep being related to memory processing, but also to distinct neural circuits being responsible for memory processing. Finally, the potential for memory systems to disengage implies that the interaction between memory systems, during wakefulness, is maintained by a functional rather than anatomical process, which raises the possibility that specific altering of neural function may induce disengagement during wakefulness, and so allow the processing benefits of sleep to be reaped while awake. Far from an exhaustive list, these examples and descriptions of the predictions are intended to provide a flavor of the type of hypothesis-driven research that can emerge from this framework.

## Abbreviations

EEG: electroencephalographic
M1: primary motor cortex
MTL: mediotemporal lobe
NREM: nonrapid eye movement
REM: rapid eye movement
SWS: slow-wave sleep
TMS: transcranial magnetic stimulation
